# Fatigue in Multiple Sclerosis Is Associated with Reduced Expression of Interleukin-10 and Worse Prospective Disease Activity

**DOI:** 10.3390/biomedicines10092058

**Published:** 2022-08-23

**Authors:** Luana Gilio, Fabio Buttari, Luigi Pavone, Ennio Iezzi, Giovanni Galifi, Ettore Dolcetti, Federica Azzolini, Antonio Bruno, Angela Borrelli, Marianna Storto, Roberto Furlan, Annamaria Finardi, Tatjana Pekmezovic, Jelena Drulovic, Georgia Mandolesi, Diego Fresegna, Valentina Vanni, Diego Centonze, Mario Stampanoni Bassi

**Affiliations:** 1 IRCCS Neuromed, 86077 Pozzilli, Italy; 2 Clinical Neuroimmunology Unit, Institute of Experimental Neurology (INSpe), Division of Neuroscience, San Raffaele Scientific Institute, 20132 Milan, Italy; 3 Faculty of Medicine, University of Belgrade, 11000 Belgrade, Serbia; 4 Institute of Epidemiology, Faculty of Medicine, University of Belgrade, 11000 Belgrade, Serbia; 5 Clinic of Neurology, Clinical Center of Serbia, 11000 Belgrade, Serbia; 6 Synaptic Immunopathology Lab, IRCCS San Raffaele Roma, 00163 Rome, Italy; 7 Department of Human Sciences and Quality of Life Promotion, University of Rome San Raffaele, 00166 Rome, Italy; 8 Department of Systems Medicine, Tor Vergata University, 00133 Rome, Italy

**Keywords:** relapsing-remitting multiple sclerosis (RR-MS), fatigue, inflammation, IL-10, NEDA-3, T2 lesion load

## Abstract

In multiple sclerosis (MS), fatigue is a frequent symptom that negatively affects quality of life. The pathogenesis of fatigue is multifactorial and inflammation may play a specific role. To explore the association between fatigue, central inflammation and disease course in MS in 106 relapsing-remitting (RR)-MS patients, clinical characteristics, including fatigue and mood, were explored at the time of diagnosis. NEDA (no evidence of disease activity)-3 status after one-year follow up was calculated. Cerebrospinal fluid (CSF) levels of a set of proinflammatory and anti-inflammatory molecules and peripheral blood markers of inflammation were also analyzed. MRI structural measures were explored in 35 patients. A significant negative correlation was found at diagnosis between fatigue measured with the Modified Fatigue Impact Scale (MFIS) and the CSF levels of interleukin (IL)-10. Conversely, no significant associations were found with peripheral markers of inflammation. Higher MFIS scores were associated with reduced probability to reach NEDA-3 status after 1-year follow up. Finally, T2 lesion load showed a positive correlation with MFIS scores and a negative correlation with CSF IL-10 levels at diagnosis. CSF inflammation, and particularly the reduced expression of the anti-inflammatory molecule IL-10, may exacerbate fatigue. Fatigue in MS may reflect subclinical CSF inflammation, predisposing to greater disease activity.

## 1. Introduction

Multiple sclerosis (MS) is a chronic autoimmune disease of the central nervous system (CNS) characterized by inflammation, demyelination, and neurodegeneration. The inflammatory process in MS is initiated and sustained by infiltration of autoreactive T and B lymphocytes, the activation of microglia and astrocytes, and the release of proinflammatory cytokines [1].

Fatigue which negatively affects quality of life in patients with MS is observed in all disease phenotypes [2]. It is frequently present at the time of diagnosis, representing the onset symptom [3]. The pathogenesis of fatigue in MS is thought to be multifactorial [4]. Although it has been associated with the degree of disability and structural damage, alternative mechanisms have been implicated, including dysfunction of the hypothalamic–pituitary–adrenal axis, altered brain activation, and neurotransmitter release [4,5].

Although not completely elucidated, immunological and inflammatory mechanisms may play important roles in the pathogenesis of MS fatigue [4]. Notably, fatigue is very common in patients with autoimmune diseases such as systemic lupus erythematosus and rheumatoid arthritis [6]. Moreover, increased serum levels of proinflammatory cytokines have been associated with fatigue in different autoimmune conditions [7,8,9] and have been demonstrated in chronic fatigue syndrome [7]. Previous studies have reported some relationship between fatigue and peripheral inflammatory biomarkers. In particular, in MS patients with fatigue, higher production capacity, increased mRNA expression, and serum levels of specific inflammatory molecules such as IL-6 and TNF have been reported [8,10,11]. However, the association between fatigue in MS and peripheral inflammatory molecules is still unclear; in fact, some investigations have not reported significant associations, and most studies have been conducted on small samples [12,13,14,15,16]. Assessment of intrathecal cytokine levels may help to identify markers of fatigue directly related to MS-specific mechanisms [17]. In addition, cerebrospinal fluid (CSF) cytokines represent a reliable marker of prospective disease activity in MS [18]. It is worthy of note that associations have been reported between fatigue and worse MS course [19,20], suggesting that higher levels of fatigue could characterize a subgroup of patients prone to develop greater disease activity. Therefore, studying the association between fatigue and CSF inflammation could be extremely useful to identify reliable biomarkers to predict disease course and tailor treatment from the time of diagnosis.

In this paper, we explored in a group of relapsing-remitting (RR)-MS patients whether fatigue assessed at diagnosis is associated with specific clinical features, and especially with a particular cytokine profile in the CSF. Additionally, we examined whether fatigue at the time of diagnosis could be a factor that predicts no evidence of disease activity (NEDA)-3, after a one-year follow-up.

## 2. Materials and Methods

### 2.1. RR-MS Patients

We retrospectively analyzed the data of 106 consecutive MS patients enrolled between 2018 and 2020. Patients were admitted to the neurology unit of Neuromed hospital (Pozzilli, Italy) and later diagnosed as suffering from RR-MS, based on clinical, laboratory and MRI parameters according to McDonald’s diagnostic criteria [21]. The study, performed in accordance with the Declaration of Helsinki, was approved by the Ethics Committee of Neuromed hospital (CE numbers 06/17 and 11/17). All patients gave written informed consent to participate in the study and to give their demographic and clinical data and biological material for scientific research. Inclusion criteria were the established RR-MS diagnosis and the ability to provide written informed consent to the study. Patients with different autoimmune and inflammatory disease and with clinically relevant medical or surgical conditions which might affect fatigue were excluded. No patient had a major psychiatric diagnosis. At the diagnosis, demographic and clinical characteristics were recorded, including disease duration, clinical disability evaluated with the Expanded Disability Status Scale (EDSS) [22], and body mass index (BMI) calculated as weight (kg)/height (m^2^). Fatigue, depression, and anxiety were also evaluated at the time of diagnosis in all patients. Fatigue was measured with the Modified Fatigue Impact Scale (MFIS), a 21-item questionnaire measuring the physical, cognitive, and psychosocial impact of fatigue (maximum score of 84) [23]. In line with a previous study, we used a cut-off of 38 for MFIS scores [24], and clinically significant fatigue was evidenced. The Beck Depression Inventory-Second Edition (BDI-II) was used to assess depressive symptoms [25]. Anxiety was measured with the State-Trait Anxiety Inventory (STAI) form Y (STAI-Y) [26].

Patients were followed up for one year. NEDA status was calculated for each patient at the end of the follow up period. NEDA was defined as the absence of radiological and clinical disease activity and the absence of disability progression (NEDA-3) [27].

Patients had not previously received either corticosteroids or immunoactive therapies. Disease modifying therapies (DMT) were initiated after diagnosis as follows: I line (interferon beta-1a 22 mcg = one patient; peginterferon beta-1a = one patient; teriflunomide = three patients; glatiramer acetate = 12 patients; dimethyl fumarate = 57 patients), II line (ocrelizumab = four patients; cladribine = five patients; fingolimod = eight patients; natalizumab = 10 patients). DMTs were selected according to practical guidelines [28] by neurologists blinded to MFIS values and CSF cytokines levels.

### 2.2. MRI Examination

Brain and spine MRI was performed at diagnosis, six months and one year after diagnosis. Additional MRI scans were performed if clinical relapses occurred. MRI included dual-echo proton density sequences, FLAIR, T1-weighted spin-echo (SE), T2-weighted fast SE, and contrast-enhanced T1-weighted SE after intravenous gadolinium (Gd) infusion (0.2 mL/kg), performed on 1.5- or 3.0-Tesla scanners. Radiological disease activity was defined as the presence of a gadolinium-enhancing (Gd+) lesion on MRI scans performed at diagnosis.

In 35 patients, cortical thickness and T2 lesion load were analyzed at diagnosis. All the images were acquired using the 3T MR scanner (GE Signa HDxt, GE Healthcare, Milwaukee, WI, USA). We used a 3D Spoiled Gradient Recalled (SPGR) T1-weighted sequence (178 contiguous sagittal slices, voxel size 1 × 1 × 1 mm^3^, TR 7 ms, TE 2.856 ms, inversion time 450 ms) and a 3D FLAIR sequence (208 contiguous sagittal 1.6 mm slices, voxel size, 0.8 × 0.8 × 0.8 mm^3^, TR 6000 ms, TE 139.45 ms; inversion time 1827 ms). We first segmented white matter lesions from FLAIR and T1 images by using the lesion growth algorithm as implemented in version 2.0.15 of the lesion segmentation tool (www.statistical-modelling.de/lst.html (accessed on 14 May 2022)) for SPM12 (https://www.fl.ion.ucl.ac.uk/spm (accessed on 14 May 2022)). Furthermore, we used the computational anatomy toolbox (CAT12, version 916, https://dbm.neuro.uni-jena.de/cat/ (accessed on 14 May 2022)) as implemented in SPM12 to extract individual cortical thickness values from lesion-filled MR images. Finally, T2 lesion load was computed from 3D T1 and 3d FLAIR images by using a well-established pipeline.

### 2.3. CSF Collection and Analysis

In all patients, CSF was collected at diagnosis by lumbar puncture. Lactate levels and oligoclonal bands (OCB) were assessed. After withdrawal, CSF was centrifuged and stored at −80 °C. Proinflammatory and anti-inflammatory molecules were analyzed using a Bio-Plex multiplex cytokine assay (Bio-Rad Laboratories, Hercules, CA, USA). All samples were analyzed in triplicate. Concentrations were expressed as picograms/milliliter. The CSF molecules examined included interleukin (IL)-1β, IL-2, IL-4, IL-5, IL-6, IL-7, IL-8, IL-9, IL-10, IL-12, IL-13, IL-17, IL-1receptor antagonist (ra), granulocyte colony-stimulating factor (G-CSF), granulocyte-macrophage colony-stimulating factor (GM-CSF), interferon (IFN)γ, tumor necrosis factor (TNF), monocyte chemoattractant protein 1 (MCP-1)/CCL2, macrophage inflammatory protein (MIP)-1α/CCL3, and MIP-1 β/CCL4.

### 2.4. Blood Collection and Analysis of Peripheral Inflammatory Biomarkers

Blood samples were collected at diagnosis. Erythrocyte sedimentation rate (ESR) and fibrinogen levels were measured. In addition, total white blood cells (WBC), neutrophils, lymphocytes and platelets counts were assessed. The neutrophil-lymphocytes ratio (NRL) was calculated as the quotient between the neutrophils and lymphocytes [29].

### 2.5. Statistical Analysis

A Kolmogorov–Smirnov test was used to assess the normality distribution of variables. Data were shown as median (interquartile range, IQR). Categorical variables were presented as number (N) and frequency (%). A *p* value ≤ 0.05 was considered statistically significant. Spearman’s nonparametric correlation was used to explore associations between not normally distributed variables. A Benjamini-Hockberg (B-H) test was applied to control the false discovery rate and the Type I errors (false positives) when analyzing correlations between MFIS and CSF inflammatory molecules. A box plot was used to depict statistically significant differences between groups. 

Logistic regression analysis was undertaken after one year (yes/no) with NEDA-3 as the dependent variable. All variables with significance levels ≥0.05 by univariate analysis were included in the multivariate models (method: enter).

Logistic regression analysis was used to explore the association between fatigue, as measured by MFIS, and reaching NEDA-3 after one year, after adjustment by multiple potential confounding factors.

All analyses were performed using IBM SPSS Statistics for Windows (IBM Corp., Armonk, NY, USA).

Missing data: disease duration in 3/106 patients (2.83%); radiological activity in 5/106 patients (4.71%); OCB in 2/106 patients (1.89%); CSF lactate in 2/106 patients (1.89%); BMI in 4/106 patients (3.77%); BDI-II, STAI-Y state/trait in 6/106 patients (5.66%); ESR in 1/106 patients (0.94%); fibrinogen in 2/106 patients (1.89%); WBC, lymphocytes, neutrophils, and NLR in 3/106 patients (2.83%).

## 3. Results

### 3.1. Fatigue and Clinical Characteristics at the Time of MS Diagnosis

The clinical characteristics of MS patients are shown in Table 1.

The MFIS total score showed strong positive correlations with the three subscales: MFIS-physical, MFIS-cognitive, and MFIS-psychosocial (all Spearman’s r ≥ 0.80, all *p* < 0.0001).

Using a cut-off of 38 for psychosocial scores, clinically significant fatigue was evidenced in 23/106 individuals (21.7%) of the MS cohort.

We explored the correlations between fatigue and other clinical characteristics at the time of diagnosis. A strong correlation was found between MFIS and mood scales. In particular, positive correlations emerged with BDI-II (Spearman’s r = 0.539, *p* < 0.0001), STAI-Y state (Spearman’s r = 0.347, *p* < 0.001) and STAI-Y trait (Spearman’s r = 0.491, *p* < 0.0001). No significant correlations were found between MFIS and the other clinical characteristics explored (data not presented).

The subgroups qualified for I and II line DMT differed neither in the baseline MFIS total score (*p* = 0.182), nor in any of its domains (MFIS physical *p* = 0.252; MFIS cognitive *p* = 0.135; MFIS psychosocial *p* = 0.796).

### 3.2. Fatigue Is Associated with Reduced IL-10 CSF Expression

We explored the correlations between MFIS and the CSF levels of a set of proinflammatory and anti-inflammatory molecules at the time of MS diagnosis. 

**Figure 1:**
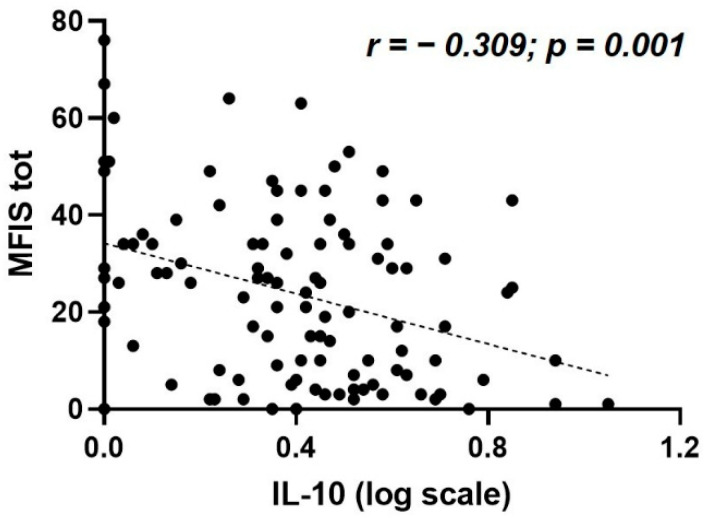
Correlation between MFIS total score and CSF IL-10 levels at MS diagnosis. Abbreviations: CSF, cerebrospinal fluid; IL, interleukin; MFIS tot, Modified Fatigue Impact Scale total score; MS, multiple sclerosis.

A significant association was found between MFIS and the CSF levels of IL-10 (Spearman’s r = −0.309, *p* = 0.001, and B-H adjusted *p* = 0.02, N = 106) (Table 2, Figure 1). The median CSF IL-10 concentration was 1.59 pg/mL (IQR 0.71–2.66). No significant correlations were found between MFIS and the other CSF molecules analyzed (Table 2). 

### 3.3. Fatigue Is not Associated with Peripheral Inflammation

We explored the association between MFIS and peripheral inflammation in MS patients. A set of peripheral blood inflammatory biomarkers has been evaluated at the time of MS diagnosis including ESR (median [IQR] = 10 [5.5–20]), fibrinogen (median [IQR] = 311 [265–352]), WBC (median [IQR] = 6.9 [5.7–8.4]), lymphocytes (median [IQR] = 1.9 [1.5–2.3]), neutrophils (median [IQR] = 4.3 [3.3–5.5]), and NLR (median [IQR] = 2.37 [1.77–2.95]).

No significant correlations were found between MFIS scores and the peripheral inflammatory biomarkers analyzed: ESR (Spearman’s r = 0.024, *p* = 0.809, N = 105), fibrinogen (Spearman’s r = −0.19, *p* = 0.849, N = 104), WBC (Spearman’s r = 0.04, *p* = 0.685, N = 103), lymphocytes (Spearman’s r = −0.039, *p* = 0.693, N = 103), neutrophils (Spearman’s r = ‒0.001, *p* = 0.992, N = 103), and NLR (Spearman’s r = 0.004, *p* = 0.971, N = 103).

### 3.4. Baseline Fatigue Predicts NEDA-3 after One Year Follow-Up 

We investigated predictive values of all variables listed in the Table 3 to reach NEDA-3 after one year in our RR-MS cohort. None of these variables was demonstrated to be statistically significant predictors, except the MFIS total score and its psychosocial domain. NEDA-3 was achieved in 61/101 (60%) of the patients independently on the type of DMTs (50/74 for the I line and 11/27 for the II line).

Results of the logistic regression analyses of the association of the MFIS total score and different domains with reaching NEDA-3 in RR-MS patients, after a one-year follow-up, are presented in Table 3. We analyzed predictive factors for reaching NEDA-3 using this variable as a dependent factor in the models. In the logistic regression analyses (dependent variable: NEDA-3, yes/no), it has been demonstrated that only MFIS psychosocial domain and total MFIS score were independent predictors of NEDA-3, after adjustment by several demographic and clinical variables at baseline: sex, age, disease duration, EDSS, radiological disease activity, total number of relapses, BDI-II, STAI-Y state, STAI-Y trait, BMI, treatment with DMTs and concentration of IL-10 in the CSF. A lower score of MFIS psychosocial domain and MFIS total score predicted higher probability of reaching NEDA-3 (OR = 0.67, 95% CI 0.49–0.90, *p* = 0.009; OR = 0.96; 95% CI 0.92–0.99; *p* = 0.039), respectively. This means that patients whose baseline score of MFIS in the psychosocial domain will increase for one point after one year, and will have a 33% lower chance of reaching NEDA-3. Additionally, those whose baseline total MFIS score will increase for one point after one year will have a 4% lower chance of reaching NEDA-3.

### 3.5. Correlations between Structural MRI Measures, Fatigue, and CSF IL-10 Levels at the Time of MS Diagnosis

Structural MRI measures were available for 35 MS patients (Supplementary Figure S1): cortical thickness (median [IQR] = 2.76 [2.65–2.83]), and T2 lesion load (median [IQR] = 0.31 [0.09–0.90]).

**Figure 2:**
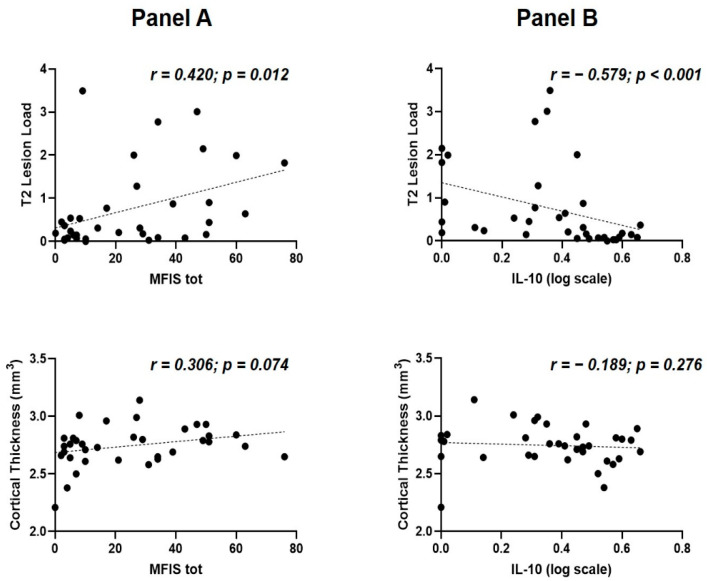
Fatigue, IL-10 and structural MRI measures at the time of MS diagnosis. (**A**) correlations between fatigue, T2 lesion load (upper) and cortical thickness (lower); (**B**) correlations between IL-10 concentrations, T2 lesion load (upper) and cortical thickness (lower). Abbreviations: IL, interleukin; MFIS tot, Modified Fatigue Impact Scale total score; MS, multiple sclerosis.

A significant correlation was found between MFIS-total and T2 lesion load (Spearman’s r = 0.420 *p* = 0.012, N = 35); conversely, no significant correlation emerged between MFIS-tot and cortical thickness (Spearman’s r = 0.306, *p* = 0.074, N = 35) (Figure 2A).

A significant correlation was also found between IL-10 and T2 lesion load (Spearman’s r = −0.579, *p* = 0.000269, N = 35). No significant association was found between the CSF levels of IL-10 and cortical thickness (Spearman’s r = −0.189, *p* = 0.276, N = 35) (Figure 2B).

## 4. Discussion

Numerous findings support the finding that activation of the immune system plays a role in the pathogenesis of fatigue; however, studies exploring the association between inflammation and fatigue in MS have yielded conflicting results [17,30]. Some association has been reported between fatigue and serum levels of the proinflammatory molecule IL-6 [12], serum levels of TNF mRNA, [10] and increased TNF and IFN𝛾 [8]. As inflammation in MS is primarily confined to the CNS compartment, inflammatory biomarkers in the CSF may provide a reliable and specific marker of fatigue in MS. After analyzing a large set of proinflammatory and anti-inflammatory CSF cytokines, we found a negative correlation between fatigue and CSF levels of the anti-inflammatory molecule IL-10 at the diagnosis. Although no association between MS fatigue and routine CSF parameters (cell count, lactate, albumin and intrathecal immunoglobulin synthesis) have been reported [30], a previous study reported a positive correlation between fatigue and levels of the proinflammatory molecule IL-6 in the CSF of patients with MS [31]. In line with our findings, an exacerbated inflammatory milieu in the CSF, resulting from the increased expression of proinflammatory cytokines or reduced levels of regulatory molecules, may characterize MS patients with fatigue. Notably, a previous study examining CSF cytokine profiles in a group of 18 patients with chronic fatigue syndrome found that IL-10 levels were significantly reduced [32]. Similarly, studies in animal models of MS (i.e., experimental autoimmune encephalomyelitis, EAE), have shown that IL-10 gene therapy has a beneficial effect on both motor and non-motor symptoms, including fatigue, anxiety and neuropathic pain [33].

IL-10 is a major anti-inflammatory cytokine and plays an important regulatory role on the inflammatory response by inhibiting the production of several proinflammatory molecules [34]. The reduced expression of IL-10 in MS patients compared to controls has been reported, particularly during disease exacerbations [35,36]. Conversely, higher levels of IL-10 have been reported in stable disease phases [37], and have been associated with reduced brain damage and disability in MS patients [38].

An altered CSF inflammatory milieu in RR-MS patients with higher levels of fatigue may be associated with increased brain damage at diagnosis and promote a worse disease course. To clarify this hypothesis, we analyzed structural MRI data available in a subgroup of 35 RR-MS patients. The negative correlation demonstrated between IL-10 CSF concentration and T2 lesion load, although limited by the low number of patients analyzed, could be consistent with the anti-inflammatory role of this molecule and might suggest that reduced CSF expression of IL-10 could be associated with increased disease activity before establishing the diagnosis of RR-MS. In addition, we found a positive correlation between MFIS and T2 lesion load in patients with RR-MS at diagnosis. An association between fatigue and white matter lesion load has been previously reported in MS [39,40]; however, other studies failed to find significant correlations [41,42]. Although differences in patients’ characteristics or MRI methodology may partly explain such discrepancies [43], it has been proposed that other factors may play a role, including lesion location, alterations in normal-appearing white matter [44], or global and regional atrophy [39,40,44]. It is conceivable that the reduced expression of anti-inflammatory molecules and the ensuing brain damage may determine higher levels of fatigue at diagnosis.

When analyzing the impact of fatigue on disease activity, we found that lower MFIS psychosocial domain and MFIS total predicted higher probability of reaching NEDA-3 (*p* = 0.009 and *p* = 0.039, respectively). In particular, patients whose baseline total MFIS score will increase for one point after one year, and will have a 4% lower chance of reaching NEDA-3. These findings were independent of mood alterations, which was consistent with previous studies [19,20] which showed that fatigue at diagnosis predicted disability progression in RR-MS and was associated with a higher risk of conversion in patients with CIS. The association between MFIS psychosocial subscale and NEDA-3 should be carefully interpreted, as results of the MFIS psychosocial subscale could be affected by possible confounding factors [23,45]. However, our results are in line with the hypothesis that in patients with RR-MS, higher levels of fatigue at diagnosis might reflect a proinflammatory state of the CSF which, in turn, is associated with higher brain damage and greater prospective disease activity.

Fatigue in MS is a complex phenomenon, and understanding the pathogenesis of this severely disabling symptom is of paramount importance to design effective therapeutic strategies to improve patients’ quality of life. In our RR-MS cohort, the prevalence of fatigue at the time of diagnosis was 21.7%. Previous studies using a similar MFIS cut-off value for fatigue have found higher proportions [24]. To explain this discrepancy, it should be noted that age at diagnosis, disease duration and disability were lower in our study; moreover, only patients with RR-MS were included.

Although it is likely that EDSS score might influence the probability of achieving NEDA-3 status, in our study, which included patients at diagnosis with mild disability, NEDA-3 after one year of follow-up was not predicted by EDSS score. The observation that other predictors of disease activity analyzed were also not associated with NEDA-3, highlights the need to study longer follow-ups in a larger number of patients even with more severe or progressive MS. The relatively short follow up duration and the lack of a healthy controls group represent major limitations of the present study. Further studies exploring fatigue fluctuations over time are important to assess the relationship between perceived fatigue and ongoing disease activity. Moreover, identifying specific CSF cytokine clusters may help to better explain the possible synergistic effect of the different molecules. Although no significant correlations emerged between fatigue and the peripheral inflammatory markers explored (ESR, fibrinogen, WBC, neutrophils, lymphocytes, NLR), the lack of peripheral cytokine levels in our study, which could have allowed simultaneous assessment of serum and CSF cytokine profiles, cannot help disentangle the differential contribution of peripheral and central inflammatory mechanisms in MS-related fatigue. Finally, studies with structural MRI measures involving a larger population are crucial to better define the influence of both IL-10 CSF levels and fatigue at diagnosis on disease activity and progression.

In conclusion, our findings could potentially implicate that self-reported fatigue at MS onset may reflect the specific manifestation of a subclinical neuroinflammatory state, predisposing a worse disease outcome.

## Figures and Tables

**Table 1:** Clinical characteristics at MS diagnosis.

		RR-MS
RR-MS patients	N	106
Age, years	Median [IQR]	34.02 [25.44–45.23]
Sex, F	N (%)	70/106 (66)
BMI	Median [IQR]	25.04 [22.43–28.65]
Disease duration, months	Median [IQR]	5.7 [1.9–24.5]
EDSS at diagnosis	Median [IQR]	1.5 [1–2.125]
Radiological activity, presence	N (%)	39/101 (38.6)
OCB, presence	N (%)	89/104 (85.6)
CSF lactate mmol/L	Median [IQR]	1.42 [1.3–1.6]
MFIS_total	Median [IQR]	24 [6.75–34]
BDI-II	Median [IQR]	7 [2.25–11.75]
STAI-Y state	Median [IQR]	41 [33.25–49.75]
STAI-Y trait	Median [IQR]	34 [30–45]

Abbreviations: BDI-II, Beck Depression Inventory-Second Edition; BMI, body mass index; CSF, cerebrospinal fluid; EDSS, Expanded Disability Status Scale; MFIS, Modified Fatigue Impact Scale; MS, multiple sclerosis; OCB, oligoclonal bands; RR-MS, relapsing remitting-MS; STAI-Y, State-Trait Anxiety Inventory-form Y.

**Table 2:** Correlations between MFIS and CSF inflammatory molecules at MS diagnosis.

CSF Molecules	Spearman’s r	*p*	B-H Adjusted *p*
IL-1β	−0.159	0.103	0.515
IL-2	−0.041	0.674	0.949
IL-4	−0.006	0.952	0.952
IL-5	−0.027	0.786	0.949
IL-6	−0.012	0.902	0.949
IL-7	−0.058	0.556	0.927
IL-8	−0.113	0.248	0.792
IL-9	−0.179	0.066	0.440
IL-10	**−0.309**	0.001	**0.020**
IL-12	0.026	0.791	0.949
IL-13	−0.088	0.370	0.792
IL-17	−0.083	0.396	0.792
TNF	−0195	0.045	0.44
IFNγ	−0.013	0.893	0.949
IL1-ra	0.098	0.318	0.792
G-CSF	−0.016	0.868	0.949
GM-CSF	−0.112	0.254	0.792
MCP-1/CCL2	0.099	0.311	0.792
MIP-1 α/CCL3	0.068	0.490	0.891
MIP-1 β/CCL4	−0.023	0.812	0.949

Abbreviations: B-H, Benjamini-Hockberg; CSF, cerebrospinal fluid; G-CSF, granulocyte colony-stimulating factor; GM-CSF, granulocyte-macrophage colony-stimulating factor; IFN, interferon; IL, interleukin; IL-1ra, interleukin-1 receptor antagonist; MCP, monocyte chemoattractant protein 1; MFIS, Modified Fatigue Impact Scale; MIP, macrophage inflammatory protein; MS, multiple sclerosis; TNF, tumor necrosis factor.

**Table 3:** Logistic regression analysis of the association of the different MFIS domains and total score, with reaching NEDA-3 in RR-MS patients, after one-year follow-up. Bold values denote statistical significance.

Variable	OR	95% CI	*p*
MFIS physical	0.95	0.89–1.01	0.089
Sex	1.22	0.39–3.83	0.733
Age at diagnosis	1.01	0.96–1.06	0.708
Disease duration	0.99	0.97–1.01	0.298
EDSS at diagnosis	1.29	0.72–2.30	0.386
Radiological disease activity	2.68	0.86–8.40	0.090
Total number of relapses at diagnosis	0.80	0.35–1.84	0.602
BDI–II	1.03	0.91–1.15	0.663
STAI-Y state	1.01	0.94–1.07	0.847
STAI-Y trait	0.93	0.84–1.02	0.107
BMI	0.94	0.84–1.06	0.306
DMTs	0.32	0.09–1.10	0.070
IL-10	0.18	0.02–1.98	0.162
MFIS cognitive	0.94	0.88–1.02	0.124
Sex	1.23	0.40–3.82	0.716
Age at diagnosis	1.00	0.95–1.06	0.888
Disease duration	0.99	0.97–1.01	0.336
EDSS at diagnosis	1.31	0.74–2.34	0.353
Radiological disease activity	2.54	0.82–7.88	0.106
Total number of relapses at diagnosis	0.73	0.32–1.65	0.445
BDI-II	1.03	0.92–1.15	0.627
STAI-Y state	0.99	0.94–1.06	0.971
STAI-Y trait	0.93	0.85–1.03	0.154
BMI	0.94	0.83–1.05	0.278
DMTs	0.30	0.08–1.05	0.059
IL-10	0.25	0.02–2.48	0.235
MFIS psychosocial	0.67	0.49–0.90	**0.009**
Sex	0.93	0.28–3.13	0.912
Age at diagnosis	1.01	0.95–1.06	0.773
Disease duration	0.99	0.97–1.01	0.580
EDSS at diagnosis	1.20	0.67–2.17	0.536
Radiological disease activity	4.27	1.19–15.32	0.026
Total number of relapses at diagnosis	0.67	0.29–1.56	0.357
BDI-II	1.03	0.92–1.17	0.588
STAI-Y state	1.01	0.94–1.08	0.809
STAI-Y trait	0.93	0.84–1.02	0.138
BMI	0.94	0.83–1.06	0.283
DMTs	0.36	0.10–1.31	0.123
IL-10	0.12	0.01–1.42	0.093
MFIS total score	0.96	0.92–0.99	**0.039**
Sex	1.10	0.34–0.35	0.876
Age at diagnosis	1.00	0.95–1.06	0.874
Disease duration	0.99	0.971.01	0.378
EDSS at diagnosis	1.33	0.73–2.39	0.348
Radiological disease activity	2.67	0.84–8.45	0.095
Total number of relapses at diagnosis	0.72	0.31–1.66	0.435
BDI-II	1.03	0.92–1.16	0.578
STAI-Y state	1.00	0.94–1.07	0.900
STAI-Y trait	0.94	0.85–1.03	0.182
BMI	0.93	0.83–1.05	0.259
DMTs	0.30	0.09–1.07	0.064
IL-10	0.14	0.11–0.60	0.113

Abbreviations: BDI-II, Beck Depression Inventory-Second Edition; BMI, body mass index; DMTs, disease modifying therapies; EDSS, Expanded Disability Status Scale; IL, interleukin; MFIS, Modified Fatigue Impact Scale; NEDA-3, no evidence of disease activity-3; STAI-Y, State-Trait Anxiety Inventory-form Y.

## Data Availability

The data that support the fundings of this study are available from the corresponding author, upon reasonable request.

## Supplementary Materials

The following supporting information can be downloaded at https://www.mdpi.com/article/10.3390/biomedicines10092058/s1, Figure S1: Structural MRI image samples.

## Abbreviations

BDI-II: Beck Depression Inventory-Second Edition; B-H: Benjamini-Hockberg; BMI: body mass index; CNS: central nervous system; CSF: cerebrospinal fluid; DMT: Disease modifying therapies; EDSS: Expanded Disability Status Scale; ESR: Erythrocyte sedimentation rate; G-CSF: granulocyte colony-stimulating factor; Gd+: gadolinium-enhancing; GM-CSF: granulocyte-macrophage colony-stimulating factor; IFN: interferon; IL: interleukin; IL1-ra: IL-1receptor antagonist; IQR: interquartile range; MCP-1: monocyte chemoattractant protein 1; MFIS: Modified Fatigue Impact Scale; MIP: macrophage inflammatory protein; MS: multiple sclerosis; N: number; NEDA: no evidence of disease activity; NRL: Neutrophil-lymphocytes ratio; OCB: oligoclonal bands; PRO: patient-reported outcomes; RR-MS: relapsing-remitting multiple sclerosis; STAI-Y: State-Trait Anxiety Inventory-form Y; TNF: tumor necrosis factor; WBC: total white blood cells; %: frequency.

## References

[1] Lassmann H., Bradl M. (2017). Multiple sclerosis: Experimental models and reality. Acta Neuropathol..

[2] Herring T.E., Alschuler K.N., Knowles L.M., Phillips K.M., Morean W.M., Turner A.P., Ehde D.M. (2021). Differences in correlates of fatigue between relapsing and progressive forms of multiple sclerosis. Mult. Scler. Relat. Disord..

[3] Patrick E., Christodoulou C., Krupp L., New York State MS Consortium (2009). Longitudinal correlates of fatigue in multiple sclerosis. Mult. Scler..

[4] Manjaly Z.M., Harrison N.A., Critchley H.D., Do C.T., Stefanics G., Wenderoth N., Lutterotti A., Müller A., Stephan K.E. (2019). Pathophysiological and cognitive mechanisms of fatigue in multiple sclerosis. J. Neurol. Neurosurg. Psychiatry.

[5] Dantzer R., Heijnen C.J., Kavelaars A., Laye S., Capuron L. (2014). The neuroimmune basis of fatigue. Trends Neurosci..

[6] Ramsey-Goldman R., Rothrock N. (2010). Fatigue in systemic lupus erythematosus and rheumatoid arthritis. PM R.

[7] Morris G., Maes M. (2014). Mitochondrial dysfunctions in myalgic encephalomyelitis/chronic fatigue syndrome explained by activated immuno-inflammatory, oxidative and nitrosative stress pathways. Metab. Brain Dis..

[8] Heesen C., Nawrath L., Reich C., Bauer N., Schulz K.H., Gold S.M. (2006). Fatigue in multiple sclerosis: An example of cytokine mediated sickness behaviour?. J. Neurol. Neurosurg. Psychiatry.

[9] Lee Y.C., Frits M.L., Iannaccone C.K., Weinblatt M.E., Shadick N.A., Williams D.A., Cui J. (2014). Subgrouping of patients with rheumatoid arthritis based on pain, fatigue, inflammation, and psychosocial factors. Arthritis Rheumatol..

[10] Flachenecker P., Bihler I., Weber F., Gottschalk M., Toyka K.V., Rieckmann P. (2004). Cytokine mRNA expression in patients with multiple sclerosis and fatigue. Mult. Scler..

[11] Alvarenga-Filho H., Salles M., Hygino J., Ferreira T.B., Sacramento P.M., Monteiro C., Vasconcelos C.C., Alvarenga R.M., Bento C.A. (2017). Fatigue favors in vitro Th1 and Th17-like cell expansion and reduces corticoid sensitivity in MS patients. J. Neuroimmunol..

[12] Malekzadeh A., Van de Geer-Peeters W., De Groot V., Teunissen C.E., Beckerman H., TREFAMS-ACE Study Group (2015). Fatigue in patients with multiple sclerosis: Is it related to pro- and anti-inflammatory cytokines?. Dis. Markers.

[13] Akcali A., Zengin F., Aksoy S.N., Zengin O. (2017). Fatigue in Multiple Sclerosis: Is it related to cytokines and hypothalamic-pituitary-adrenal axis?. Mult. Scler. Relat. Disord..

[14] Iriarte J., Subirá M.L., Castro P. (2000). Modalities of fatigue in multiple sclerosis: Correlation with clinical and biological factors. Mult. Scler..

[15] Giovannoni G., Thompson A.J., Miller D.H., Thompson E.J. (2001). Fatigue is not associated with raised inflammatory markers in multiple sclerosis. Neurology.

[16] Yaldizli O., Kumar M., Vago S., Kreuzfelder E., Limmroth V., Putzki N. (2009). Fatigue is not associated with impaired function of regulatory T cells in untreated patients with multiple sclerosis. Eur. Neurol..

[17] Patejdl R., Penner I.K., Noack T.K., Zettl U. (2016). Multiple sclerosis and fatigue: A review on the contribution of inflammation and immune-mediated neurodegeneration. Autoimmun. Rev..

[18] Stampanoni Bassi M., Iezzi E., Landi D., Monteleone F., Gilio L., Simonelli I., Musella A., Mandolesi G., De Vito F., Furlan R. (2018). Delayed treatment of MS is associated with high CSF levels of IL-6 and IL-8 and worse future disease course. J. Neurol..

[19] Runia T.F., Jafari N., Siepman D.A., Hintzen R.Q. (2015). Fatigue at time of CIS is an independent predictor of a subsequent diagnosis of multiple sclerosis. J. Neurol. Neurosurg. Psychiatry.

[20] Cavallari M., Palotai M., Glanz B.I., Egorova S., Prieto J.C., Healy B.C., Chitnis T., Guttmann C.R. (2016). Fatigue predicts disease worsening in relapsing-remitting multiple sclerosis patients. Mult. Scler..

[21] Thompson A.J., Banwell B.L., Barkhof F., Carroll W.M., Coetzee T., Comi G., Correale J., Fazekas F., Filippi M., Freedman M.S. (2018). Diagnosis of multiple sclerosis: 2017 revisions of the McDonald criteria. Lancet Neurol..

[22] Kurtzke J.F. (1983). Rating neurologic impairment in multiple sclerosis: An expanded disability status scale (EDSS). Neurology.

[23] Kos D., Kerckhofs E., Carrea I., Verza R., Ramos M., Jansa J. (2005). Evaluation of the Modified Fatigue Impact Scale in four different European countries. Mult. Scler..

[24] Flachenecker P., Kümpfel T., Kallmann B., Gottschalk M., Grauer O., Rieckmann P., Trenkwalder C., Toyka K.V. (2002). Fatigue in multiple sclerosis: A comparison of different rating scales and correlation to clinical parameters. Mult. Scler..

[25] Sica C., Ghisi M., Lange M.A. (2007). The Italian versions of the Beck Anxiety Inventory and the Beck Depression Inventory-II: Psychometric properties and discriminant power. Leading-Edge Psychological Tests and Testing Research.

[26] Pedrabissi L., Santinello M. (1989). Manuale Dell’adattamento Italiano dello STAI di Spielberger, Forma Y.

[27] Havrdova E., Galetta S., Hutchinson M., Stefoski D., Bates D., Polman C.H., O’Connor P.W., Giovannoni G., Phillips J.T., Lublin F.D. (2009). Effect of natalizumab on clinical and radiological disease activity in multiple sclerosis: A retrospective analysis of the Natalizumab Safety and Efficacy in Relapsing-Remitting Multiple Sclerosis (AFFIRM) study. Lancet Neurol..

[28] (2019). Practice guideline recommendations summary: Disease-modifying therapies for adults with multiple sclerosis: Report of the Guideline Development, Dissemination, and Implementation Subcommittee of the American Academy of Neurology. Neurology.

[29] Hasselbalch I.C., Søndergaard H.B., Koch-Henriksen N., Olsson A., Ullum H., Sellebjerg F., Oturai A.B. (2018). The neutrophil-to-lymphocyte ratio is associated with multiple sclerosis. Mult. Scler. J. Exp. Transl. Clin..

[30] Biberacher V., Schmidt P., Selter R.C., Pernpeinter V., Kowarik M.C., Knier B., Buck D., Hoshi M.M., Korn T., Berthele A. (2018). Fatigue in multiple sclerosis: Associations with clinical, MRI and CSF parameters. Mult. Scler..

[31] Brenner P., Granqvist M., Königsson J., Al Nimer F., Piehl F., Jokinen J. (2018). Depression and fatigue in multiple sclerosis: Relation to exposure to violence and cerebrospinal fluid immunomarkers. Psychoneuroendocrinology.

[32] Peterson D., Brenu E.W., Gottschalk G., Ramos S., Nguyen T., Staines D., Marshall-Gradisnik S. (2015). Cytokines in the cerebrospinal fluids of patients with chronic fatigue syndrome/myalgic encephalomyelitis. Mediat. Inflamm..

[33] Grace P.M., Loram L.C., Christianson J.P., Strand K.A., Flyer-Adams J.G., Penzkover K.R., Forsayeth J.R., van Dam A.M., Mahoney M.J., Maier S.F. (2017). Behavioral assessment of neuropathic pain, fatigue, and anxiety in experimental autoimmune encephalomyelitis (EAE) and attenuation by interleukin-10 gene therapy. Brain Behav. Immun..

[34] Kwilasz A.J., Grace P.M., Serbedzija P., Maier S.F., Watkins L.R. (2015). The therapeutic potential of interleukin-10 in neuroimmune diseases. Neuropharmacology.

[35] Van Boxel-Dezaire A.H., Hoff S.C., van Oosten B.W., Verweij C.L., Dräger A.M., Adèr H.J., van Houwelingen J.C., Barkhof F., Polman C.H., Nagelkerken L. (1999). Decreased interleukin-10 and increased interleukin-12p40 mRNA are associated with disease activity and characterize different disease stages in multiple sclerosis. Ann. Neurol..

[36] Inogés S., Merino J., Bandrés E., De Castro P., Subirá M.L., Sánchez-Ibarrola A. (1999). Cytokine flow cytometry differentiates the clinical status of multiple sclerosis (MS) patients. Clin. Exp. Immunol..

[37] Waubant E., Gee L., Bacchetti P., Sloan R., Cotleur A., Rudick R., Goodkin D. (2001). Relationship between serum levels of IL-10, MRI activity and interferon beta-1a therapy in patients with relapsing remitting MS. J. Neuroimmunol..

[38] Petereit H.F., Pukrop R., Fazekas F., Bamborschke S.U., Röpele S., Kölmel H.W., Merkelbach S., Japp G., Jongen P.J., Hartung H.P. (2003). Low interleukin-10 production is associated with higher disability and MRI lesion load in secondary progressive multiple sclerosis. J. Neurol. Sci..

[39] Tedeschi G., Dinacci D., Lavorgna L., Prinster A., Savettieri G., Quattrone A., Livrea P., Messina C., Reggio A., Servillo G. (2007). Correlation between fatigue and brain atrophy and lesion load in multiple sclerosis patients independent of disability. J. Neurol. Sci..

[40] Sepulcre J., Masdeu J.C., Goñi J., Arrondo G., Vélez de Mendizábal N., Bejarano B., Villoslada P. (2009). Fatigue in multiple sclerosis is associated with the disruption of frontal and parietal pathways. Mult. Scler..

[41] Bakshi R., Miletich R.S., Henschel K., Shaikh Z.A., Janardhan V., Wasay M., Stengel L.M., Ekes R., Kinkel P.R. (1999). Fatigue in multiple sclerosis: Cross-sectional correlation with brain MRI findings in 71 patients. Neurology.

[42] Papadopoulou A., Müller-Lenke N., Naegelin Y., Kalt G., Bendfeldt K., Kuster P., Stoecklin M., Gass A., Sprenger T., Radue E.W. (2013). Contribution of cortical and white matter lesions to cognitive impairment in multiple sclerosis. Mult. Scler..

[43] Popescu V., Schoonheim M.M., Versteeg A., Chaturvedi N., Jonker M., Xavier de Menezes R., Gallindo Garre F., Uitdehaag B.M., Barkhof F., Vrenken H. (2016). Grey Matter Atrophy in Multiple Sclerosis: Clinical Interpretation Depends on Choice of Analysis Method. PLoS ONE.

[44] Rocca M.A., Parisi L., Pagani E., Copetti M., Rodegher M., Colombo B., Comi G., Falini A., Filippi M. (2014). Regional but not global brain damage contributes to fatigue in multiple sclerosis. Radiology.

[45] Larson R.D. (2013). Psychometric properties of the modified fatigue impact scale. Int. J. MS Care.

