# Supplementary material for: Fatigue in Multiple Sclerosis Is Associated with Reduced Expression of Interleukin-10 and Worse Prospective Disease Activity
Source: Biomedicines. 2022 Aug 23;10(9):2058. doi: 10.3390/biomedicines10092058 (PMC9495727; doi:10.3390/biomedicines10092058)
Supplement: Supplementary file 1 [file biomedicines-10-02058-s001.zip › biomedicines-1782854-supplementary.pdf]

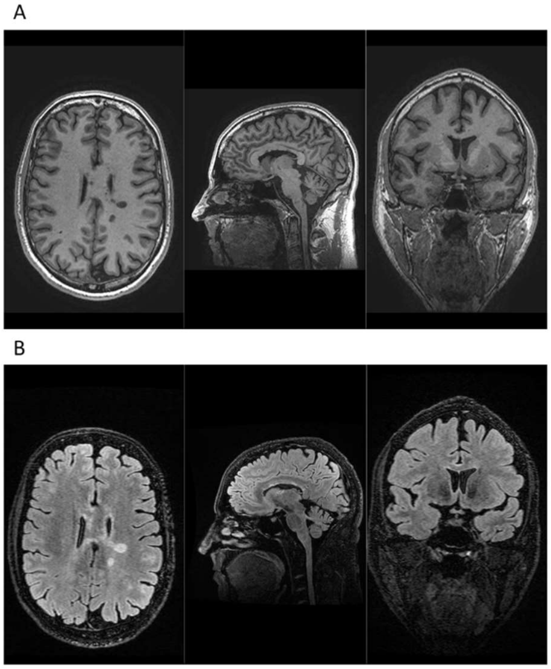

Supplementary Figure S1:

Figure Legends

A: high-resolution 3D T1 MRI image sample used to estimate cortical thickness.

B: high-resolution 3D T2 FLAIR CUBE MRI image used to estimate lesion burden.
